# High-Speed Scalable Silicon-MoS_2_ P-N Heterojunction Photodetectors

**DOI:** 10.1038/srep44243

**Published:** 2017-03-10

**Authors:** Veerendra Dhyani, Samaresh Das

**Affiliations:** 1 Centre for Applied Research in Electronics, Indian Institute of Technology Delhi, New Delhi-110016, India

## Abstract

Two-dimensional molybdenum disulfide (MoS_2_) is a promising material for ultrasensitive photodetector owing to its favourable band gap and high absorption coefficient. However, their commercial applications are limited by the lack of high quality p-n junction and large wafer scale fabrication process. A high speed Si/MoS_2_ p-n heterojunction photodetector with simple and CMOS compatible approach has been reported here. The large area MoS_2_ thin film on silicon platform has been synthesized by sulfurization of RF-sputtered MoO_3_ films. The fabricated molecular layers of MoS_2_ on silicon offers high responsivity up to 8.75 A/W (at 580 nm and 3 V bias) with ultra-fast response of 10 μsec (rise time). Transient measurements of Si/MoS_2_ heterojunction under the modulated light reveal that the devices can function up to 50 kHz. The Si/MoS_2_ heterojunction is found to be sensitive to broadband wavelengths ranging from visible to near-infrared light with maximum detectivity up to ≈1.4 × 10^12^ Jones (2 V bias). Reproducible low dark current and high responsivity from over 20 devices in the same wafer has been measured. Additionally, the MoS_2_/Si photodetectors exhibit excellent stability in ambient atmosphere.

Transition metal dichalcogenides (TMDs) are predicted to be promising candidate for next generation electronics^1^,^2^,^3^, optoelectronics^4^,^5^, and sensor^6^ devices. The intrinsic nature of carriers in two dimensional semiconductors offers numerous advantages such as high mobility, high sensitivity, fast response and chemical stability^7^,^8^,^9^,^10^. Among several of the layered TMDs, MoS_2_ has attracted great interest because of its distinctive electronic, optical, and catalytic properties^4^,^5^,^6^,^9^,^10^,^11^,^12^. In the recent time a lot of efforts have been devoted to demonstrate the potential of MoS_2_ for a variety of applications^13^,^14^,^15^. MoS_2_ has been used as an active layer in high mobility transistors (70–100 cm^2^/V-s) with very high on/off ratio of 10^8 ^16,17. More importantly, the broad spectrum from which MoS_2_ can absorb light i. e. visible to near-infrared spectral region (350–950 nm) is higher than GaAs and Si (one order of magnitude in a thickness of less than 1 nm) which makes it an ideal material for photonic devices^18^,^19^. However, the use of MoS_2_ for practical applications is still limited due to the lack of scalability in its fabrication process. Several methods such as mechanical/chemical exfoliation, chemical vapour deposition etc^20^,^21^,^22^,^23^. that have been reported are mired with drawbacks. In the exfoliation method, poor control over thickness and limited size of MoS_2_ films are the main deterrents. On the other hand there are reports available, where large-area MoS_2_ layers has been directly obtained using chemical vapour deposition and solution processed methods^4^,^24^,^25^. The absence of high quality p–n junctions also limits the performance of the MoS_2_ based devices. Although photodetectors based on mono/multilayer MoS_2_ heterostructure have shown excellent device characteristics in terms of high sensitivity (up to 7 A/W) and wide band response^26^,^27^,^28^,^29^ their applicability still suffers due to complex fabrication process.

In this work, we report Si/MoS_2_ (p-n) heterojunction based photodetector contrived from a very simple, reproducible and scalable fabrication process. To fabricate the heterojunction, we have adopted CVD process for the growth of MoS_2_ thin film as mentioned in previously published reports^24^,^26^. The durability and scalability of the devices examined after two months are found to remains intact. The fabricated heterojunction exhibited excellent photoresponse properties in the visible to near-infrared wavelengths with a maximum responsivity of 8.75A W^−1^ and fast rise time of 10 μs. The excellent performance of multilayered MoS_2_ heterostructure may lead the way for the fabrication of high speed photodetectors.

## Experimental details

### Synthesis of large area MoS_2_ thin film

In the present study, MoS_2_ thin films were fabricated by the sulphurization of molybdenum trioxide (MoO_3_) in N_2_ ambient at low temperature. In a typical process, a 10 nm thick film of MoO_3_ was first deposited on the SiO_2_/Si substrates by reactive sputtering (in presence of Ar and O_2_ with 1:1 ratio). The MoO_3_/SiO_2_/Si samples were then placed at the centre of a tube furnace preheated to 500 °C and maintained for 20 min. Sulphur powder was introduced in the upstream zone of the furnace, where temperature is around 200 °C. As a result of the sulphurization process, thin films of MoS_2_ with large area coverage were grown. The structural and morphological properties of deposited samples were characterized by Scanning electron microscopy (SEM- Ziess evo 18), and atomic force microscopy (AFM- Bruker dimension). In order to confirm the formation of MoS_2_ Raman spectroscopy (Horiba Raman Lab RAM HR 800 Evolution) has been employed.

### Device fabrication and measurements

**Figure 1:**
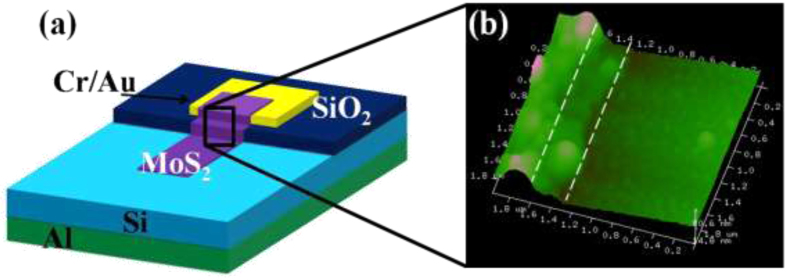
Schematic representations of the Si/MoS_2_ p-n heterojunction photodetector, (**b**) AFM image of MoS_2_ layer at metal contact and heterojunction interface.

Si/MoS_2_ heterojunction photodetectors were fabricated using C-MOS compatible technology. P-type silicon (100) with a thermally grown silicon oxide (SiO_2_) of 100 nm was used as the starting wafer for the fabrication of Si/MoS_2_ heterojunction. To expose the silicon, SiO_2_ was selectively etched after the first UV-lithography step. A two step photo-lithography process has been used to make the heterojunction. In the first step, squares of dimension 100 μm^2^ were opened in SiO_2_, which define the Si/MoS_2_ junction area. The metal contact (Cr/Au) has been patterned in the second lithography process. The detailed fabrication process is given in the supplementary information (Fig. S1). On the top of this structure, thin layer of MoO_3_ (10 nm) was deposited by reactive sputtering. Samples were cleaned in the oxygen plasma for 30 sec before the deposition of MoO_3_. After the deposition of MoO_3_, MoS_2_ was grown via sulphurization process discussed earlier. Cr/Au layers on the top serve as the one electrode for the electrical measurements, while a 100 nm thick layer of Al has been deposited on the back side of the silicon for second electrode. Figure 1(a) shows a typical schematic representation of the device structure. MoS_2_ thin film on Si and metal contact can be seen in the AFM image Fig. 1(b). In order to measure the photoresponse of Au-MoS_2_-Au metal-semiconductor-metal (MSM) photodetector was fabricated. The fabrication process was the same as Si/MoS_2_ heterojunction, except that the bottom oxide was not etched. Electrical measurements of the device were performed using Keithley semiconductor analyzer (SCS4200) under ambient conditions. The spectral response of the photodetector has been measured in the wavelength range 300 nm to 1100 nm.

## Results and Discussion

**Figure 2:**
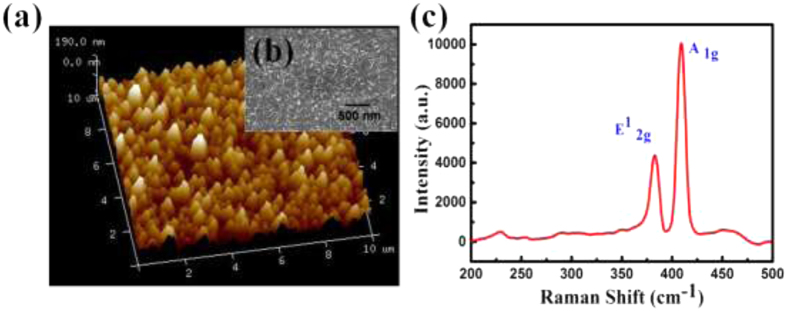
(**a**) AFM image and (**b**) Raman spectra of as synthesized MoS_2_.

The morphology of the as deposited MoS_2_ film surface observed under an atomic force microscopy (AFM) is shown in Fig. 2(a). The surface of the film seems to be composed of vertically aligned nanoflakes with average width between 20–40 nm. It is likely that these stratified features in the MoS_2_ thin film formed as a result of the difference in the thermal expansion coefficient of substrate and the MoS_2_ film during sulfurization. The growth of MoS_2_ film was homogenous and it covered the entire substrate surface (~2inch) uniformly. Similar growth has been observed by Ellmer *et al*.^30^ and S. B, Sadale *et al*.^31^ in case of WS_2_. A Scanning electron microscope (SEM) image of the as synthesized MoS_2_ is shown in the inset of Fig. 2(a). The above SEM image confirms clearly the formation of nanoflakes of MoS_2_. Similar morphology of MoS_2_ surfaces has also been observed on SiO_2_ substrate (Fig. S2(b) supplementary information). It is believed that this morphology originates from the hexagonal basal plane symmetry of the MoS_2_ crystal itself, suggesting that the layers grown are oriented along c-axis of the MoS_2_ unit cell^24^,^32^. A crystallinity of the MoS_2_ thin film samples were examined using Raman spectroscopy (514 nm laser and 10 mW power). The characteristic Raman peaks for the E^1^_2g_ and A_1g_ vibration modes appeared at 384 cm^−1^ and 408 cm^−1^ corresponding to MoS_2_ hexagonal phase, as shown in Fig. 2(c)^24^,^32^. The overall intensity of the peaks and the relative ratio, of the E^1^ 2 g, the A_1_g peaks were similar to that from high quality exfoliated MoS_2_. The crystallinity of the MoS_2_ thin film was further investigated by XRD spectra (shown in Fig. S2(c) supplementary information). The preferred direction of growth was found to be along the c-axis (0001) of hexagonal phase^24^,^32^,^33^. This suggests that these films were of good structural quality.

**Figure 3:**
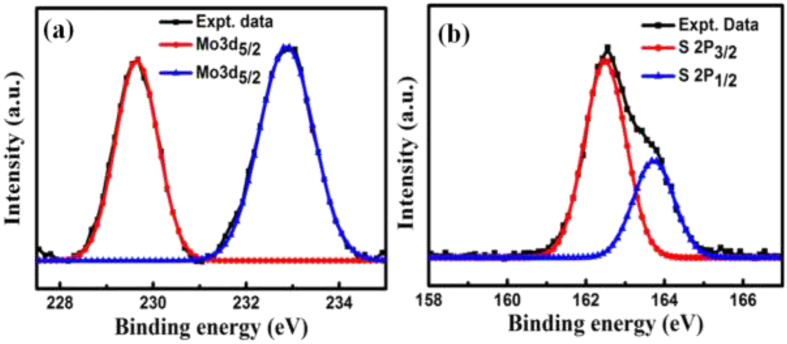
(**a**) and XPS spectra showing the binding energy of (**a**) Mo 3d and (**b**) S 2p electron.

The chemical bonding and electronic properties of the MoS_2_ films were investigated by X-ray photoelectron spectroscopy (XPS) using Al–K_α_ radiation of energy 1486.6 eV. Binding energies of Mo 3d and S 2p electrons are shown in Fig. 3(a,b). Two distinct peaks for Mo 3d electrons observed at 229.4 and 232.6 eV were attributed to 3d_5/2_ and 3d_3/2_ chemical states, respectively^34^,^35^,^36^,^37^,^38^. The possibility of Mo-silicide or migration of Mo in Si was investigated by comparing the XPS peaks of Mo and Si with that reported in literature. The silicide related peaks of Mo are reported at lower binding energies for 3d_5/2_ and 3d_3/2_^35^,^36^. Similarly the XPS peak of Si 2P_3/2_ was observed at 99.4 eV, which gets shifted towards lower binding energy for the Mo-silicide formation. In our samples the peak position of Si 2P_3/2_ has been observed at 102.3 eV^35^,^36^ (Fig. S2(a) in supplementary information). XPS analysis for both of the Mo and Si clearly confirmed that formation of silicide or migration of Mo in Si has not occurred in our samples. The spectrum for S 2p electrons is resolved into Gaussian peaks located at 162.5 eV and 163.8 eV, owing to S 2p_3/2_ and S 2p_1/2_ states, respectively. The observed peak positions of spin-orbit coupled Mo 3d and S 2p electrons are in excellent agreement with those reported in literature^33^, confirming the synthesis of pure hexagonal 2-H phase MoS_2_.

**Figure 4:**
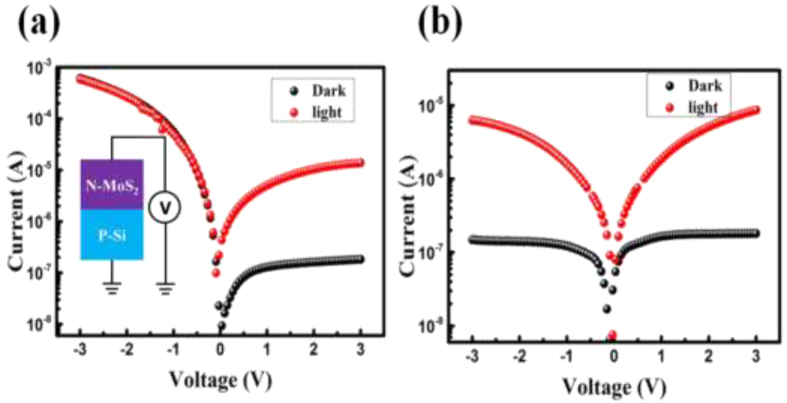
I-V characteristics of (**a**) Si-MoS_2_ p-n heterojunction and (**b**) Au/MoS_2_/Au MSM photodetector.

In general bulk MoS_2_ exhibit n-type conductivity and makes a type –II p-n junction with the p-Si. Since the Raman spectra from the as-synthesized MoS_2_ sample shows a splitting of more than 25 cm^−1^ between E^1^_2g_ (384 cm^−1^) and A_1g_ (408 cm^−1^) modes, which is similar to bulk (multilayered) structure^34^,^36^,^37^,^38^,^39^. A typical current-voltage (I–V) characteristic of the Si/MoS_2_ heterojunction under the light and dark condition is depicted in Fig. 4(a). During the measurement, the p-type substrate was kept at ground and the bias voltage was applied to the top MoS_2_ contact and therefore a negative voltage to the top contact would make the junction forward biased. The voltage biasing scheme is shown in the inset of Fig. 4(a). The high asymmetry observed in the I–V characteristics clearly indicates the formation of a very good p-n junction between the n-type MoS_2_ and p-type Si. A dark current of ~1.8 × 10^−7^was measured at reverse bias of 3 V, which enhances ~127 times upon illumination under 560 nm light of intensity 5 mW/cm^2^. In order to estimate the ideality factor η of the fabricated heterojunction, the semi-log J-V plot has been fitted according to the following equation^18^,^34^.


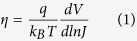


Here q is the unit charge, k_B_ is the Boltzmann’s constant, and J is current density. Current density (J) versus voltage (V) plot of the n-MoS_2_ and p-Si heterojunction is given in the supplementary information (Fig. S3(a)). The diode ideality factor is found to be ~2.4. Since no passivation layer exists at the MoS_2_-Si interface, the relatively large ideality factor might have originated due to the existence of interface defects states^34^. Furthermore, the I-V curve of the heterojunction in the dark could be described by thermionic emission theory of charge carriers over a potential zero bias barrier Φ_b_, from the Si to MoS_2_. Φ_b_ can be defined by the following equation^18^.


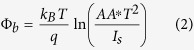


Here A is the area (100 × 100 μm^2^) of the device, A* (32 A cm^−2^K^−2^ for p-Si) is the effective Richardson constant. Based on the above equations, Φ_b_ was estimated to be approx. 0.545 eV. The C–V measurement on this heterojunction has been carried out and has been added as Fig. S3(b) in supplementary information. The used p-silicon wafers were boron doped with ~10^15^ cm^−3^. From the C–V analysis the doping concentration for MoS_2_ of ~2 × 10^16^ cm^−3^ was extracted for C-V analysis. Photoresponse of MoS_2_ MSM photodetector was measured under the same illumination level to compare the performance with the MoS_2_-Si p-n junction. Figure 4(b) shows the IV characteristics of Au- MoS_2_-Au MSM photodetector. The measured dark current for MSM photodetector was found to be ~2.5 × 10^−7^ (at reverse bias of 3 V). Upon illumination (560 nm) a photo-to-dark current ratio of ~72 is achieved. For a better comparison MSM photodetector with one side Au and one side ohmic (low work function metal Ag) contact was made. I-V characteristic of Au-MoS_2_-Ag is shown in Fig. S4(a). Semi ohmic nature (low rectification ratio of ~4) indicates the low barrier height of Au-MoS_2_ contact^39^. Additionally the photocurrent the two MSM photodetectors are not much different from each other (Fig. (S4(b)). From these results it can be easily noted that the MoS_2_-Si heterojunction possess larger gain than the MoS_2_ MSM photodetector. The high rectification ratio, low turn-on voltage, and small ideality factor coupled with high photoresponse clearly demonstrate the high quality of the fabricated heterojunction photodetector.

**Figure 5:**
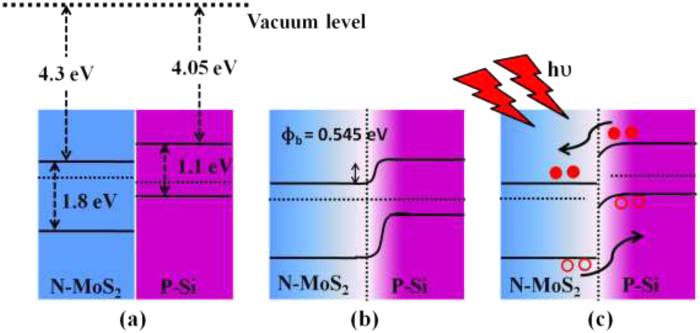
(**a**) Band edge alignment of Si/MoS_2_ heterostructure and band diagram of MoS_2_-Si heterojunction (**b**) at 0 V bias (**c**) at reverse bias.

The conduction mechanisms behind the operation of Si/MoS_2_ heterojunction photodetectors can be explained using the help of energy band diagrams, as shown in Fig. 5. The alignment of the conduction and valence band edges of Si and MoS_2_ in proper energy scale, is shown in Fig. 5(a). Under an equilibrium condition (zero bias), the band diagram of the MoS_2_-Si heterojunction would be as shown in Fig. 5(b), where an electron affinity of 4.3 and a band gap of 1.8 for MoS_2_ was assumed^34^,^40^. Since the thickness of MoS_2_ layer is around 4–6 nmit would be fully depleted. As a result no bend bending in the energy band diagram in MoS_2_ is expected as shown in Fig. 5(b). When the junction is formed between p-type Si with a work function (Φ Si) of 4.9 eV and n-type MoS_2_ (Φ MoS_2_ ~ 4.7 eV)^34^, a large barrier potential would be created at the MoS_2_/Si interface. On applying a reverse bias, the E_f_ (p-Si) is lifted to higher values, as shown in Fig. 5(c). That in turn enhances the electric field across the depletion region gradually and results in the expansion of barrier potential as well. This will open up a large number of accessible states for the holes to be injected into Si region. Here the higher carrier mobility of MoS_2_ also helped to enhance the photoresponse of the photodetector. Under light illumination, electron–hole pairs are generated, which are then separated by this large barrier potential and collected by the electrodes. As a result of that photocurrent, which was totally suppressed near V ≈ 0, significantly improves under small reverse bias.

Next, the photoresponse from Si/MoS_2_ heterojunction and MoS_2_ MSM devices were measured within the wavelength range 350 nm to 1050 nm. Spectral responsivity of a photodetector is directly proportional to the internal gain and it represents the efficiency of a detector for the specific optical signals. Responsivity of a photodetector can be defined as follows^40^,^41^,^42^,^43^.


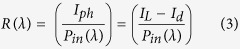


**Figure 6:**
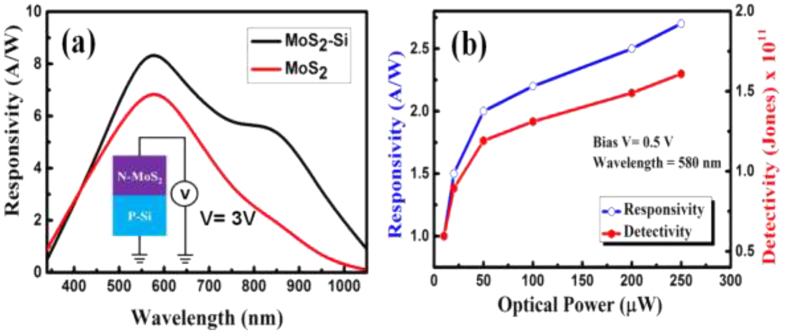
(**a**) responsivity of Si/MoS_2_ heterojunction and MoS_2_ MSM photodetector (inste shows the biasing condition for Si/MoS_2_ heterojunction) and (**b**) optical power density dependent responsivity and detectivity of Si/MoS_2_ heterojunction photodetector.

Here I_L_ and I_d_ are the current under light and dark condition, while P_in_ is the incident optical power density. Figure 6(a) shows the spectral response from the MoS_2_/Si heterojunction and MoS_2_ MSM device at a bias of 3 V. Boththe devices register a wide spectral response, for wavelength ranging from 450 nm–1000 nm. Two prominent peaks centered around 580 nm and 860 nm were observed for Si/MoS_2_ heterojunction. On the other hand, for MoS_2_ MSM photodetector, a single the peak was observed at 580 nm. It can therefore be concluded that the high responsivity at 580 nm is due to the absorption of MoS_2_ film in the visible region^34^. The other peak at a wavelength of 860 nm corresponds to conventional Si based photodiode, which was observed from the absorption of the Si substrate^42^. As can be seen from Fig. 6(a), the responsivity for the MoS_2_ MSM photodetector in the longer wavelength range (greater than 600 nm) is relatively lower than Si/MoS_2_ heterojunction, due to the absence of Si junction. The improved photoresponse from MoS_2_-Si device can be explained as follows. The introduction of the p-n heterojunction in the MoS_2_/Si photodetector leads to the formation of an inherent large barrier height (depletion region) which gets further enhanced under a reverse bias. Under light illumination, electron–hole pairs are generated, which then gets separated by the large electric field and are collected at electrodes. The performance MoS_2_/Si heterojunction photodetector also has contribution from the surface morphologies of deposited MoS_2_ film. The photo-carrier generation and collection efficiency might have been enhanced due light trapping phenomena and high photosensitivity from the textured feature (nanostructures) at the surface. The high mobility of carrier in MoS_2_-Si p-n junction due to high electric field might be another of the contributing factor. The value of responsivity for MoS_2_-Si and MoS_2_ MSM photodetector as shown in Fig. 6(a) measured at 580 nm are 8.50 A/W and 6.8 A/W respectively. It is known that high reflection losses reduces the responsivity at smaller wavelengths, while for the longer wavelengths, reduction in responsivity is caused by band gap limit. Apart from responsivity (R), the performance of any photodetector can be expressed by detectivity (D*). These two characteristics properties are related to each other according to following equation^4^,^18^,^34^.


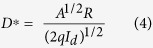


Detectivity represents the ability of a detector to detect weak optical signals, which has been calculated from the above equation. It is assumed that the dark current is dominated by the shot noise for estimating detectivity. The other sources of noise (Johnson and flicker noise) are mainly thermal fluctuations, which can be neglected. Equation (4) suggests that a low dark current and a high responsivity are desired for the photodetector to exhibit high detectivity. The peak detectivity of the fabricated p-n heterojunction diode is estimated to be ~2 × 10^11^ cm-Hz^1/2^. W^−1^ (or Jones) at 0.5 V bias, which get enhanced up to ~1.4 × 10^12^ Jones for 2 V bias. High detectivity indicates that the MoS_2_-Si heterojunction is extremely sensitive to small optical input signals. The order of detectivity found in this work is high compare to other reported MoS_2_-based photodetectors^4^,^7^,^18^,^34^. Most importantly, the high D* is acquired at small external bias voltage (0.5 V), in contrast to the relatively high bias voltage needed in previous works^4^,^18^,^34^. Figure 6(b) shows the linear increase of both responsivity and detectivity for low power illumination, and a a sub-linear dependence at higher power levels. The increase of illumination intensity leads to the enhanced electron–hole pair generation rate, resulting in a higher photocurrent. Under weak light intensity, the photogenerated electrons in MoS_2_ will be captured by the trap states. As a result of reduced recombination, the lifetime for the photogenerated holes can be greatly prolonged, leading to higher R and D*. However, the available states will be remarkably reduced with increasing light intensity, eventually causing the saturation of photoresponse^34^. The sub-linear behaviour of the responsivity and detectivity (Fig. 6(b)) indicates that trap states in the MoS_2_ layer or at the junction interface is responsible for this phenomenon.

**Figure 7:**
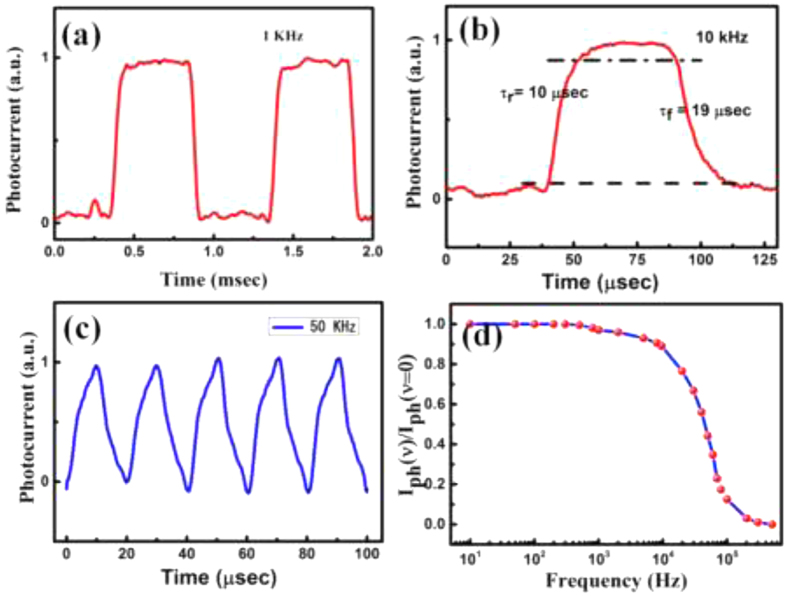
Time response characteristics under modulated light (**a**) 1 kHz, (**b**) 10 kHz (magnified), (**c**) 50 kHz and (**d**) Frequency dependence of normalized relative change in photocurrent (Iph(υ)/Iph(0)).

Transient measurements of Si/MoS_2_ junction under different illumination level are shown in Fig. S4 (supplementary information). The steep rise and fall edges suggest a fast response speed, indicating that electron–hole pairs could be effectively generated and separated in the Si/MoS_2_ heterojunction. To further investigate the response time of MoS_2_-Si heterojunction, transient measurements were performed under the illumination of modulating light source. A modulated laser source of wavelength 660 nm was used for these experiments. Response of the fabricated devices has been extracted in terms of the voltage across a resistance of 100 KΩ connected to the device (in series). The schematic representations of measurement setup and time response characteristics (for 10 kHz modulation) are given in the supplementary information (Fig. S4). The measurements have been carried out with a modulation up to 50 kHz of laser source. Figure 7(a) shows the time response of Si/MoS_2_ photodetector under the 1 kHz modulated light. In the time domain, the speed of a PD is often characterized by the rise time (τ_r_, the time interval from10% to 90% of the maximum photocurrent) and the fall time (τ_f_, the time interval from 90% to 10% of the maximum photocurrent) of its response to an impulse signal. The magnified plot of time response for one cycle is shown in Fig. 7(b). Further analysis reveals a small τ_r_ of 10 μs, as well as a small τ_f_ of 19 μs. The values obtained here are remarkably high and much faster than any other reported MoS_2_-based PDs Time response of the Si/MoS_2_ heterojunction for 50 kHz laser modulation is also shown inset of Fig. 7(c). The high performance of the photodetector could be ascribed to the following aspects. First, the existence of a strong build-in electric field at the MoS_2_ and Si interface can greatly facilitate the separation and transport of photo-generated carriers. This leads to the large response and relatively high speed of the heterojunction. Second aspects is the high in-plane mobility of carriers in the MoS_2_ layer, which offers high speed paths for the transport of photogenerated carriers in this vertically standing layered structure. For 100 K ohm load resistance the cut off frequency obtained from the following eq. (5) should be 33.3 kHz.


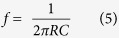


This high cut off frequency reflects in the high speed operation of the present device. The relative change in the current (I_ph(υ)_) of the photocurrent as a function of frequency in the range from 10 to 500 kHz is shown in Fig. 7(d). The relative change (defined as I_ph(υ)_ = I_L_ − I_d_)of the photocurrent only decreases by 50% at a high frequency of ≈40 kHz, implying that the Si/MoS_2_ based PDs can operate at much higher frequencies. A comparison of improved performances of our Si/MoS_2_ heterostructure has been given in Table 1 with other MoS_2_ based photodetectors.

**Figure 8:**
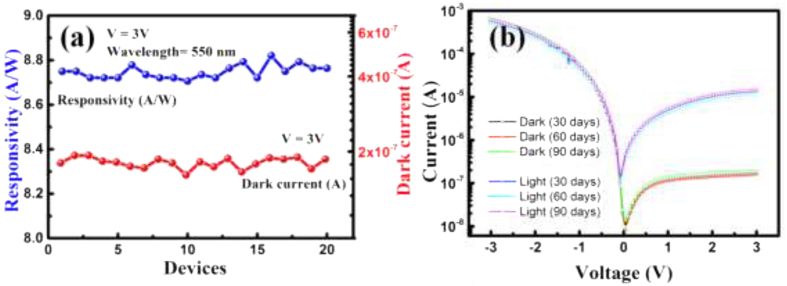
Scalability and stability test. (**a**) responsivity and dark current of 20 devices fabricated in simultaneously on single substrate, (**b**) I-V characteristics of Si/MoS_2_ photodetector for different time period.

The scalability of the process has also been investigated. Twenty devices has been fabricated and tested on the same substrate. The responsivity and dark current variation of these devices are shown in Fig. 8(a). A negligible differences in the responsivity and dark current shows the reproducibility of the measurements, which reflects in the high scalability of the fabrication process. Similarly the scalability of Au-MoS_2_-Au MSM photodetector was also investigated. The responsivity and dark current measurements for the 20 devices fabricated on the same substrate are presented in supplementary information. Furthermore, we investigated the air stability of the Si/MoS_2_ photodetector by exposing it directly in air for more than three months without any encapsulation and found the similar results. Figure 8(b) shows I-V characteristics of Si/MoS_2_ under normal ambient conditions. The device has been tested for consecutive three months and shows good atmospheric stability.

## Conclusion

Si/MoS_2_ heterojunction was fabricated by depositing MoS_2_ films with a vertically standing layered structure on p-type silicon. MoS_2_ layer was grown on wafer scale using the sulphurization MoO_3_ thin films. The devices exhibit excellent photosensitive characteristics such as large responsivity (~8.75 A/W for 580 nm at 3 V bias), high detectivity (~1.4 × 10^12^ Jones for 2 V bias) and very fast time response of 10 μsec. Transient measurements reveal that our photodetector exhibits excellent stable and reproducible dynamic response upon high frequency pulsed light, enabling it suitable for high-frequency or high-speed optical- switch applications. Moreover, the combination of reproducible and scalable fabrication process used in this work should be suitable for multiplexed photodetector arrays.

## Additional Information

**How to cite this article**: Dhyani, V. and Das, S. High-Speed Scalable Silicon-MoS_2_ P-N Heterojunction Photodetectors. *Sci. Rep.* **7**, 44243; doi: 10.1038/srep44243 (2017).

**Publisher's note:** Springer Nature remains neutral with regard to jurisdictional claims in published maps and institutional affiliations.

## Supplementary Material

Supplementary Information

## Figures and Tables

**Table 1:** A Comparison of the performance of MoS_2_ and heterojunction based photodetector.

PDs structure (Device area)	Responsivity (A/W)	Response time(τr/τf)	Detectivity (Jones)	Ref.
MoS_2_ Schottky MSM PDs	1.04 A W^−1^	40/50 μs		22
Si/MoS_2_ heterojunction	7	50/50 ms	10^10^	29
MoS_2_-QD/Si heterostructure	2.8	-	0.8 × 10^12^	34
Si/MoS_2_ heterojunction (5 × 5 μm^2^)	11.9	30.5/71.6 μs	—	4
MoS_2_ Schottky MSM PDs	0.55- 1	0.2/1.7 ms	—	13
Si/MoS_2_ heterojunction (10 × 10 μm^2^)	0.210	0.3/0.3 ms	—	40
Si/MoS_2_ heterojunction	8.75 A/W	10/19 μs	1.4 × 10^12^	This Work
