# Supplementary material for: High-Speed Scalable Silicon-MoS2 P-N Heterojunction Photodetectors
Source: Sci Rep. 2017 Mar 10;7:44243. doi: 10.1038/srep44243 (PMC5345101; doi:10.1038/srep44243)
Supplement: Supplementary Information [file srep44243-s1.pdf]

High-Speed Scalable Silicon-MoS_2 P-N Heterojunction Photodetectors

Veerendra Dhyani[1], and Samaresh Das[1*]

[1]*Centre for Applied Research in Electronics, Indian Institute of Technology Delhi, New Delhi-110016, India*

Fig.S1 Schematic fabrication process of MoS2-Si p-n junction photodetector

For the fabrication of MoS_2-Si heterojunction 100 nm SiO_2 has been grown on p-Si wafer. A two step photo-lithography process was used to define the heterojunction area, in which SiO_2 was etched. Buffered oxide (buffered HF) etchant was used for the etching of SiO_2. After the etching of SiO_2, metal contacts of Cr/Au were fabricated by etching process. 10 nm thick

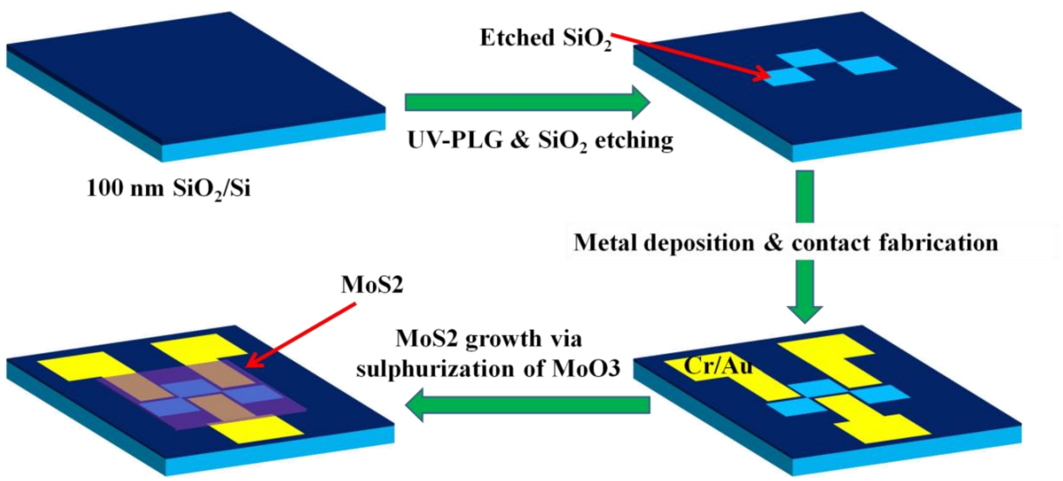

chromium (Cr) and 50 nm thick gold (Au) stacks were deposited by RF sputtering, which would be used as electrodes. A second photo-lithography process followed by wet etching of metals (Cr and Au) has been carried out after the deposition. Thin layer of MoO_3 (10 nm) was on the samples deposited by reactive sputtering. After the deposition of MoO_3 sulphurization process has been carried to synthesize MoS_2 thin film.

Structural characterization:

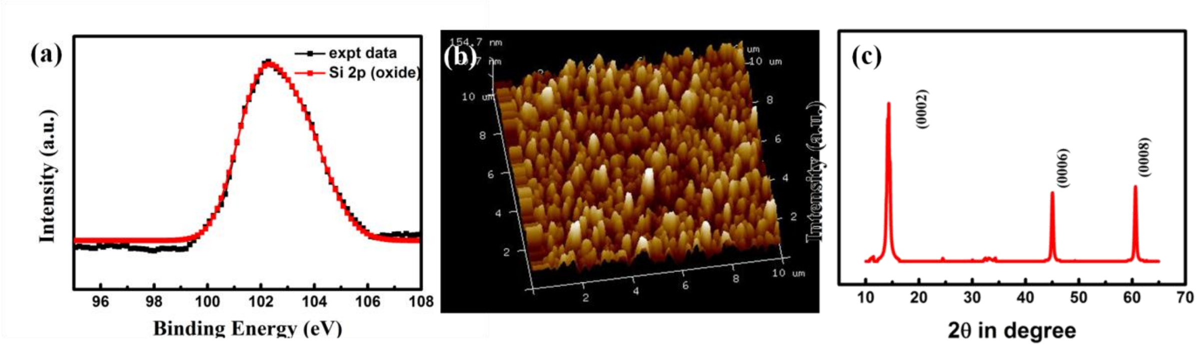

Fig. S2 (a) XPS Spectra showing the binding energy of Si (for oxide), The binding energy observed from the Fig. S2 (a) shows the oxide formation of Si. That was originated due to oxide on Si surface. Formation of Mo-silicide was not observed from the XPS analysis of Mo and Si. The morphology of MoS_2 was composed of vertically aligned nanoflakes with average width of between 20-40 nm, which remains nearly same on SiO_2 also (Fig. S2(b)). The XRD spectra (shown in the Fig (S2 (c)) shows the growth direction of MoS_2 is along (0002), (0006) and (0008) crystal planes. These peaks correspond to the c-axis growth of hexagonal planes.

J-V plot and C-V of the MoS_2-Si heterojunction

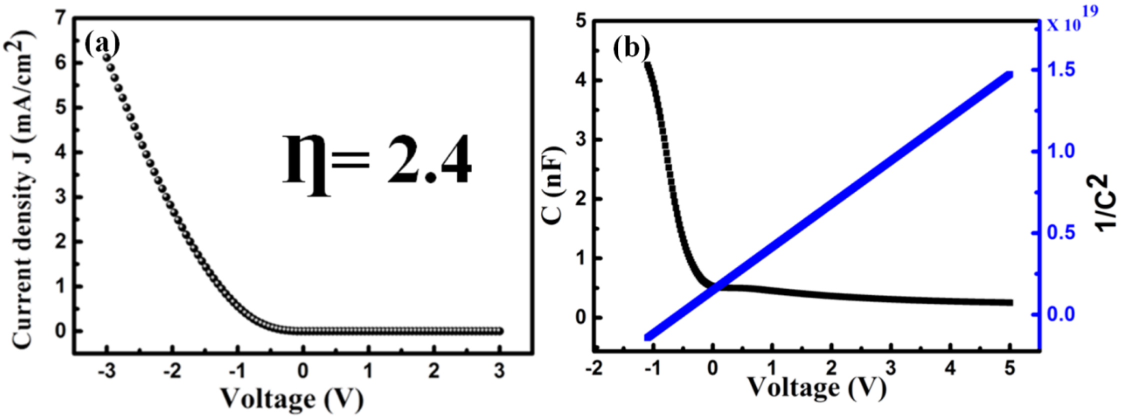

Fig. S3 (a) The extracted Ideality factor from Current density-Voltage plot was found to be ~ 2.4 for MoS_2-Si heterojunction. Fig.S3 (b) Capacitance-Voltage plot of MoS_2-Si heterojunction The C-V measurement of this heterojunction has been carried out Fig.S3 (b). The doping concentration for MoS_2 of ~$2x10^{16}$ cm^{-3} was extracted for C-V analysis. In the I-

V and C-V measurement the p-Si was kept grounded and the bias voltage was given on the top n-MoS_2 contact. Thus for a positive bias voltage the junction becomes reverse biased.

MSM device with one ohmic contact (low work function)

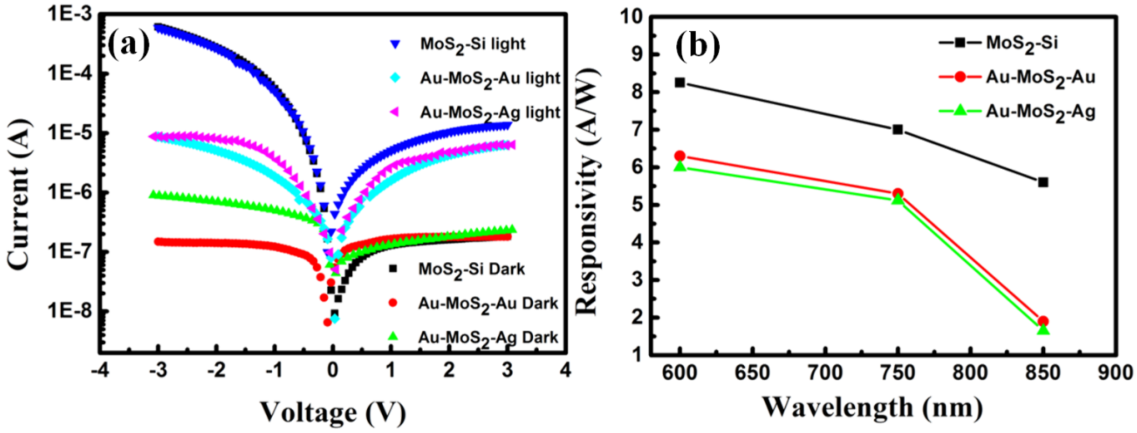

Fig. S4 (a) IV-Characteristics (dark and Light) and (b) responsivity of MoS_2-Si heterojunction, Au-MoS_2-Au MSM and Au-MoS_2-Ag MSM Photodetectors are clearly shows the high gain in MoS_2-Si heterojunction over other two MSM devices. The comparison photoresponse characteristics of Au-MoS_2-Au MSM and Au-MoS_2-Ag MSM Photodetectors with Si-MoS_2 heterojunction are shown in Fig S4. Smaller rectification in Au-MoS_2-Ag MSM device indicates lower barrier height at Au-MoS_2 interface. Also the photo-gain in the both device is nearly same. From these results it can be easily noted that the MoS_2-Si heterojunction possess larger gain than the MoS_2 MSM photodetector. For longer wavelength gain is even much higher for MoS_2-Si heterojunction compare to MSM devices.

Transient measurements

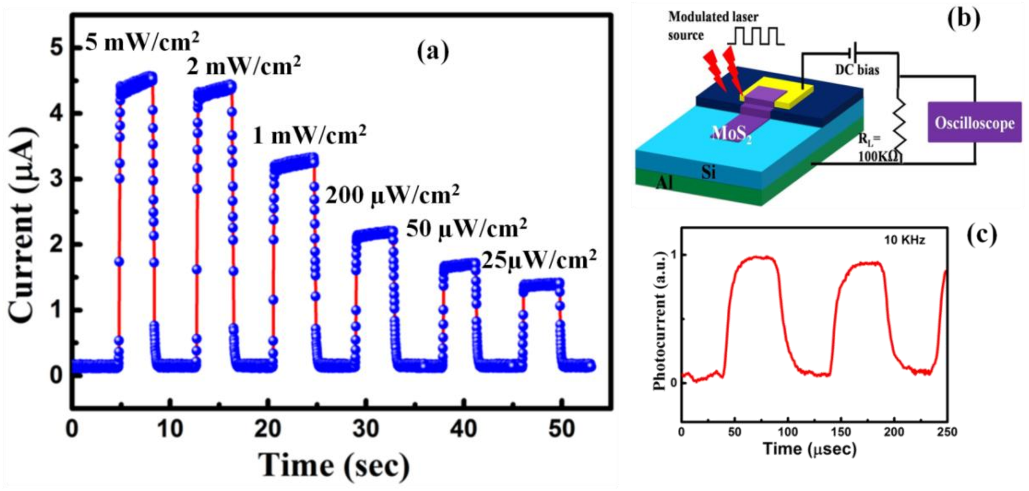

Fig. S5. (a) Transient measurement of Si/MoS_2 heterojunction photodetector under different illumination powers, (b) schematic diagram of time response measurements and (c) time response of Si/MoS_2 heterojunction photodetector under 10 kHz modulated light

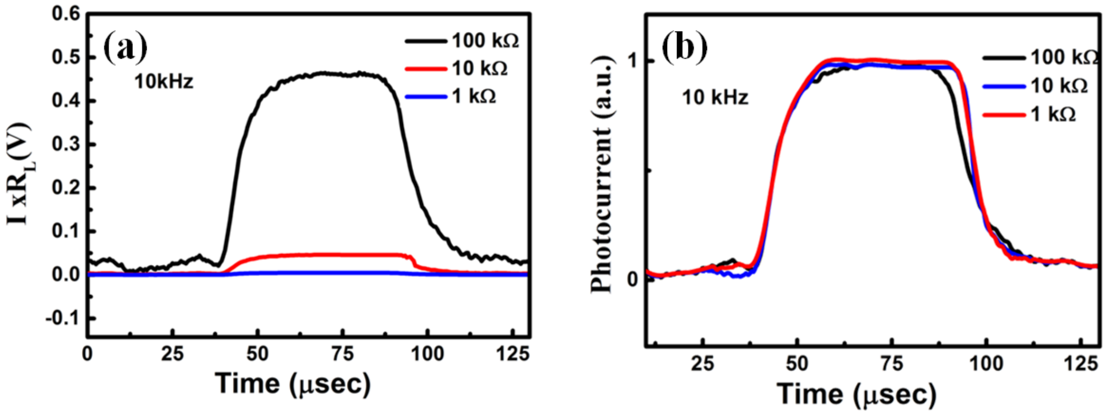

Fig. S6. Transient measurements of Si/MoS_2 heterojunction photodetector for different load resistances, Fig. (a) Shows the voltage across the resistances and (b) shows the normalized of photocurrent. Transient measurement for different load resistance shows that for smaller resistance small improvement in the response time was observed. However use of low resistance makes the signal very small, which is difficult to detect. Thus a load resistance of 100KΩ was used. Further increasing the load resistance will increase the time constant.

Scalability measurements in as Synthesized MoS_2

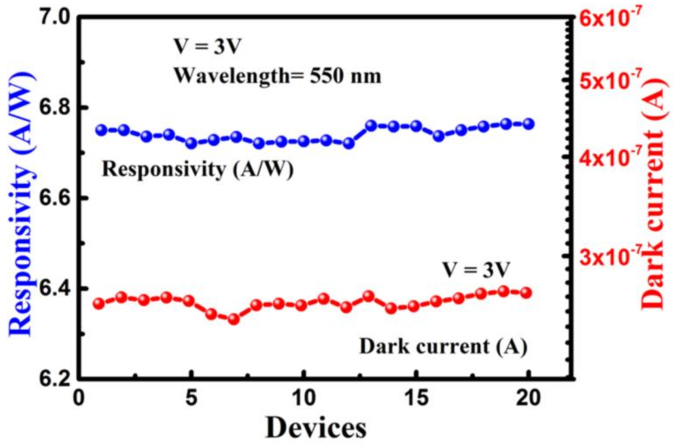

Fig. S7. The scalability of as synthesized MoS_2 the responsivity and dark current measurement for the 20 different active areas on same substrate are shown in Fig S7 (supplementary information).
